# Bronchiolitis Obliterans in an Adult Male After Admission With Panton-Valentine Leucocidin Staphylococcal Pneumonia

**DOI:** 10.7759/cureus.91611

**Published:** 2025-09-04

**Authors:** Sabha Nadeem, Georgina Sargent, Gordon Wood, Nawaid Ahmad

**Affiliations:** 1 General Medicine, The Shrewsbury and Telford Hospital NHS Trust, Telford, GBR; 2 Internal Medicine, The Shrewsbury and Telford Hospital NHS Trust, Telford, GBR; 3 Acute Medicine, The Shrewsbury and Telford Hospital NHS Trust, Telford, GBR; 4 Respiratory Medicine, The Shrewsbury and Telford Hospital NHS Trust, Shrewsbury, GBR; 5 Respiratory and Acute Medicine, The Shrewsbury and Telford Hospital NHS Trust, Telford, GBR

**Keywords:** atypical pneumonia, bronchiolitis obliterans syndrome, complicated community acquired pneumonia, obliterative bronchiolitis, panton–valentine leukocidin (pvl)-associated s. aureus infection

## Abstract

A middle-aged man with no smoking or respiratory history presented with shortness of breath and facial swelling due to influenza. His condition deteriorated rapidly, and he required intensive care admission and intubation. He was found to have Panton-Valentine leucocidin (PVL) Staphylococcal pneumonia, with bilateral pneumothorax and subcutaneous emphysema. He responded well to antibiotics and chest drainage and was subsequently discharged. His follow-up radiology initially showed almost complete resolution. However, over the course of five years, he had multiple GP attendances and hospital admissions for recurrent infections with continued breathlessness on exertion. A follow-up CT suggested features of bronchiolitis obliterans (BO), which has not been previously recognised as associated with PVL Staphylococcal infections. Specialist teams have suggested active observation with consideration of transplantation in the event of deterioration. This case demonstrates that BO can be seen with previously unrecognised infectious aetiology and should be considered in any patient with appropriate symptomatology following a severe respiratory infection.

## Introduction

Bronchiolitis obliterans (BO) is a chronic small airway obstructive lung disease and is irreversible [1]. There are three known BO entities: post-infectious (PIBO), post-lung transplantation, and post-haematopoietic stem cell transplantation (HSCT). Although these are separate, they all have similarities in histopathological characteristics and developmental pathways [1]. The presentation of BO is known to be more common in children than in adults. Here we discuss a rare case presentation of an adult male developing BO after his admission with pneumonia, caused by Panton-Valentine Leucocidin (PVL) *Staphylococcus aureus*.

## Case presentation

A middle-aged man first presented to the hospital with face and neck swelling following a viral illness. He was not known to have any respiratory disease, was a non-smoker, and did not have any known exposure to asbestos or other known causes of respiratory disease. He was diagnosed initially with pneumonia caused by influenza B; however, his condition deteriorated rapidly, and he required intubation and intensive care admission. His sputum culture tested positive for *Staphylococcus aureus*, while blood cultures showed *Staphylococcus* isolates encoding PVL genes.

**Figure 1:**
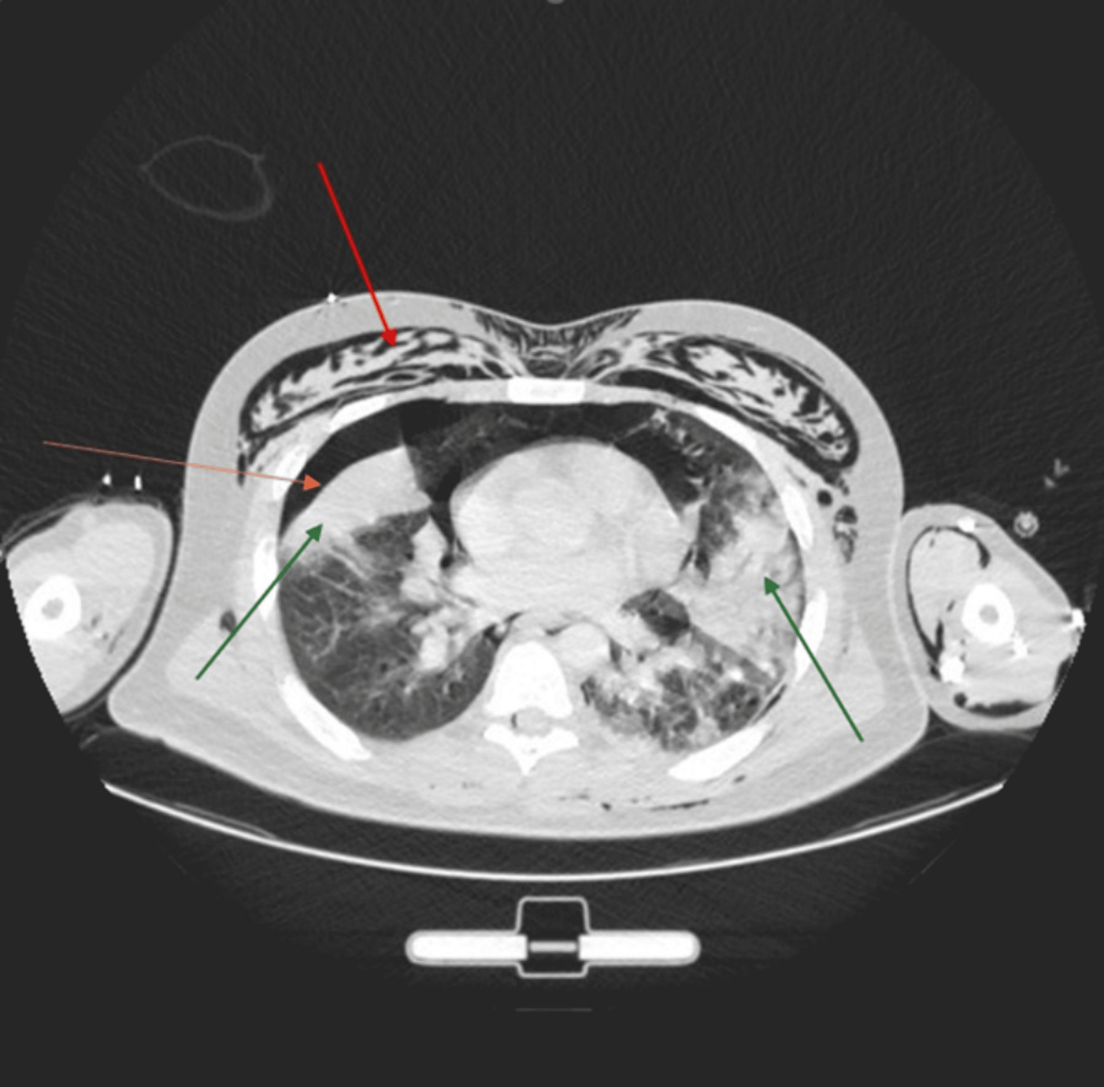
CT scan showing significant subcutaneous emphysema (red arrow), right-sided pneumothorax (orange arrow), and bilateral consolidation (green arrows).

**Figure 2:**
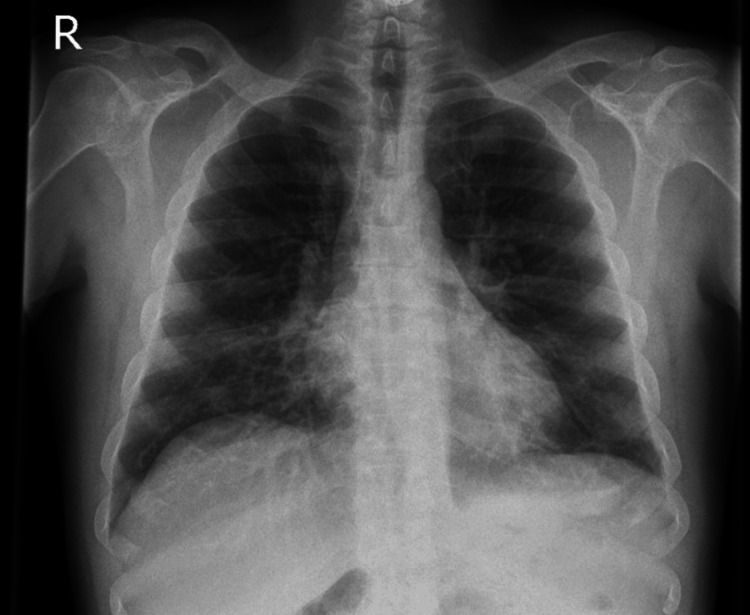
Chest X-ray showing almost complete resolution of previous changes.

A computed tomography (CT) of the thorax showed extensive bilateral consolidation scattered throughout the lungs, along with bilateral pneumothorax, pneumomediastinum, and surgical emphysema (Figure 1). Bilateral chest drains were inserted, and he responded well to linezolid. After three weeks of treatment, he was discharged. A follow-up chest X-ray performed a month later showed almost complete resolution of his previous condition (Figure 2).

He was followed up six months later in the respiratory clinic. At this time, his symptoms were minimal aside from mild exertional breathlessness and an episodic non-productive cough. A vasculitic screen and serology testing were negative (Table 1). A follow-up CT showed a post-inflammatory 3mm nodule, but otherwise showed great improvement from previous imaging. Further respiratory follow-up was delayed due to the COVID pandemic, but a follow-up CT was arranged. This did not show any further deterioration except for a small right-sided basal pleural effusion, which was too small to consider intervention.

**Table 1:** Summary of pertinent blood results ANA, antinuclear antibodies; ANCA, antineutrophil cytoplasmic antibody; HIV, human immunodeficiency virus; IgE, immunoglobulin E; IgG, immunoglobulin G; MC&S, microscopy, culture, and sensitivity; RAST, radioallergosorbent test; RSV, respiratory syncytial virus

Test	Result
Respiratory polymerase chain reaction	Positive for Influenza B, and negative for Influenza A and RSV (initial admission)
Legionella and pneumococcal antigen	Negative
Respiratory sample for MC&S: blood-tinged sputum	A heavy growth of *Staphylococcus aureus* along with *Candida albicans* (initial admission)
Blood culture	Staphylococcus aureus
Serum tryptase	Normal
HIV 1 and HIV 2 antibody	Negative
ANA, ANCA	Negative
Haemophilus antibody level	Normal
Tetanus IgG antibody	Normal
Pneumococcal IgG	Normal
Rheumatoid factor	Normal
Serum A1-antitrypsin	Normal
House dust mite RAST, cat dander (cat E1), Timothy grass RAST	0
IgE	Less than 1
Mycobacteria MC&S	Negative for acid-fast bacilli

**Figure 3:**
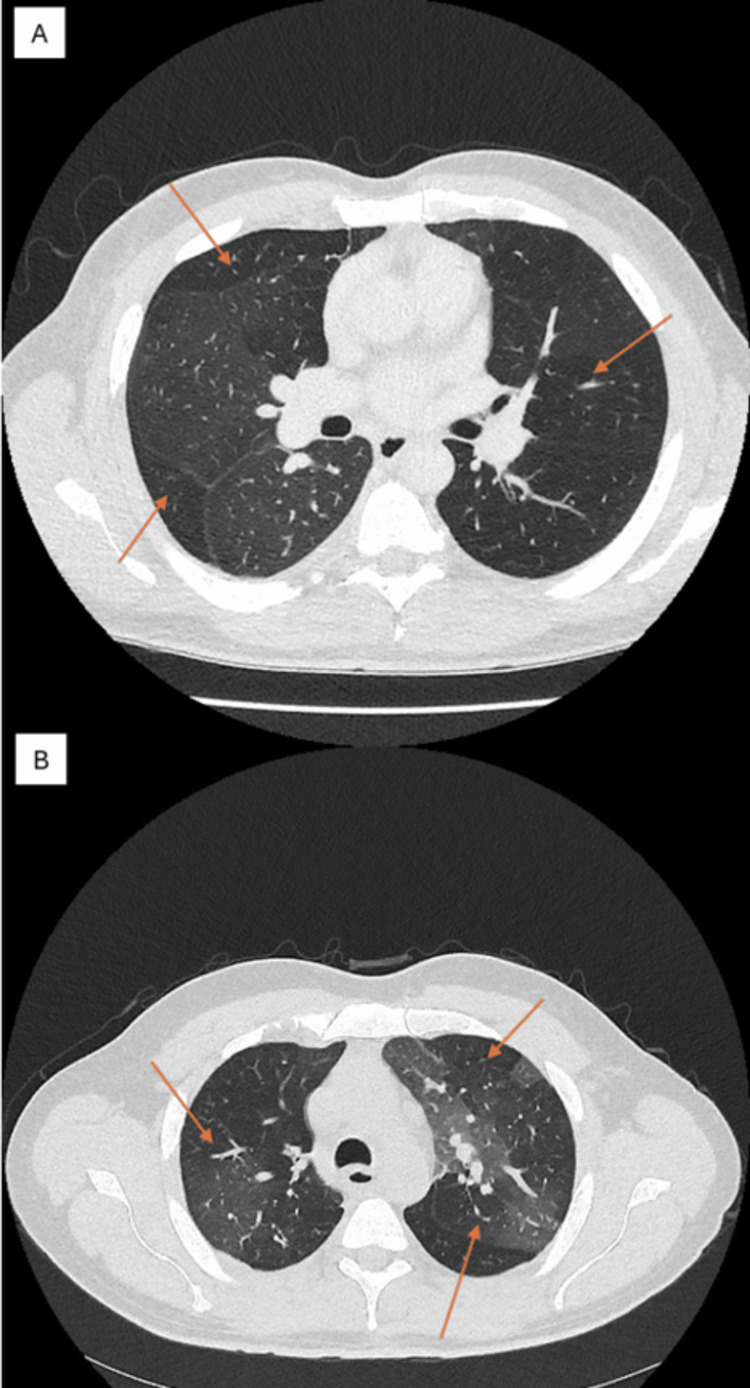
CT at diagnosis of bronchiolitis obliterans, with arrows showing air trapping and mosaic attenuation

Three years following the first episode, the patient was admitted again with community-acquired pneumonia (CAP) for three days. On this admission, CT showed features of BO. Spirometry showed a worsening obstructive picture compared to his post-discharge results (Tables 2, 3). He had another presentation with CAP the next year. Blood tests during his admission were negative for allergies, and his autoantibody screen was negative. Sputum showed no growth and was negative for acid-fast bacilli. His alpha-1-antitrypsin level and rheumatoid factor were also normal. His follow-up CT once again suggested BO (Figure 3).

**Table 2:** Spirometry results 10 weeks post-discharge FVC, forced vital capacity; FEV1, forced expiratory volume 1; TLCO, transfer factor for carbon monoxide

Spirometry	Values	% Predicted
FVC	1.86	49%
FEV1	1.3	42%
FEV1/FVC	70%	-
Peak expiratory flow	3.96	51%
TLCO	4.44	58%

**Table 3:** Spirometry at diagnosis of bronchiolitis obliterans FVC, forced vital capacity; FEV1, forced expiratory volume 1; TLCO, transfer factor for carbon monoxide

Spirometry	Values	% Predicted
FVC	1.91	57%
FEV1	1.19	43%
FEV1/FVC	62%	-
Peak expiratory flow	3.46	42%
TLCO	2.93	54%

After follow-up at the respiratory outpatient department, he was commenced on ICS/LABA combination inhaler for symptomatic relief. His predominant symptomatology was shortness of breath on exertion. He did not notice much symptomatic benefit, and thus he was upgraded to a triple therapy (LABA/LAMA/ICS) inhaler and referred to a specialist centre for consideration of lung transplant. Unfortunately, since inhalers did not improve his symptoms, they were stopped. His symptoms stabilised, and he was considered not suitable for lung transplantation at present; however, he is undertaking yearly spirometry to monitor for signs of progression, with further consideration of transplant if deterioration is noted.

## Discussion

BO (or obliterative bronchiolitis) is a subtype of bronchiolitis, defined as an irreversible clinical syndrome associated with small airways injury [2,3,4]. This is common in children, but in adults it can be secondary to infection, post-lung transplantation, or post-haematopoietic stem cell transplantation [1-3,5-8]. The histology of BO is distinctive and obliterative in nature [2-4,9]. Another related term, “bronchiolitis obliterans syndrome” (BOS), is used to denote the occurrence of an obstructive ventilatory defect after solid-organ or bone marrow transplantation mainly [3,5]. Other similar terms can be seen historically: BO had previously been divided into proliferative and constrictive, based on histological findings, with proliferative being now classed under cryptogenic organizing pneumonia (COP) [10]. COP, previously called as bronchiolitis organizing pneumonia (BOOP), is entirely different from BO in that it is more “acinar” or interstitial disease compared to BO, which is an airway disease [11]. COP can have a restrictive or mixed obstructive picture [12]. This case report and further discussion will be limited to BO.

Patients with BO typically present with progressive difficulty in breathing and cough over weeks and months, sometimes associated with wheezing but distinct from asthma episodes [2,3,13]. Abnormal pulmonary function is frequently characterized by an obstructive airflow pattern on spirometry [2,3,13]. Constrictive BO histologically shows luminal narrowing, caused by compression because of concentric fibrosis of the airway subepithilium along with granulation tissue [10]. The presence of a mosaic perfusion pattern with air trapping is a key radiological feature of BO on high-resolution CT [13]. Chest X-rays are normally unremarkable [3]. Treatments offered usually are bronchodilators and corticosteroids (inhaled and oral) [13]. Additionally, macrolides, cytotoxic agents, and other immunomodulator therapies can be trialled, but most patients with BO do not show improvement with current treatment options [13]. From 1995 to 2018, approximately 1% of primary lung transplantations were for BO [14].

BO can be caused by inhalational injury because of toxic chemicals, viral infections, systemic/autoimmune conditions, post-transplant, and idiopathic/cryptogenic [3,13,15].

Most of the studies on BO in adults refer to post-transplantation complications. However, one retrospective study by Parambil et al. [13] was a particularly insightful study about BO in a non-transplant adult population, which was conducted at Mayo Clinic from 1996 to 2003. The median adult age group was 54 years, with 69% being women with only 14% having a smoking history [13]. This identified that obstructive bronchiolar disease in the non-transplant population is commonly associated with rheumatoid arthritis, cryptogenic constrictive bronchiolitis, or other autoimmune conditions [13]. Our patient was also middle-aged, although male, and was a non-smoker. Although he did not have a background of RA, he did not respond well to treatment.

There has also been a report of a rare case of a woman in her mid-forties with a history of systemic lupus erythematosus who developed shortness of breath, productive cough, and wheezing three months after being treated for Stevens-Johnson syndrome because of second-generation cephalosporins [16]. Our patient had no background of any systemic diseases and had no Stevens-Johnson syndrome.

BO has various infectious causes. It also occurs more in children than in adults. As per Nguyen et al. [17], children with adenovirus developed post-infectious BO in a five-year period [17]. Admissions lasted nearly 30 days, and they showed poor response to bronchodilators [17]. Other causes primarily in children are measles virus or mycoplasma [3].

This case report is of a patient who had PVL *Staphylococcus aureus* infection. *Staphylococcus aureus* forms a toxin, PVL, which has two subunits (F and S) that are leucocidal and dermonecrotic together [18,19]. PVL *Staphylococcus aureus* normally causes skin infections and rarely causes necrotizing pneumonia [20]. This case is unique because it is a rare non-transplant adult presentation of BO caused by PVL *Staphylococcus aureus*. We have not been able to find any other literature demonstrating this as an aetiology.

## Conclusions

BO is a major life-altering respiratory condition that can arise from a variety of different aetiologies. Post-infectious cases are known, but no previous cases have been reported following PVL Staphylococcus aureus. This case shows that it should be considered in patients with ongoing symptoms after any severe infection, and a high-resolution CT of the thorax is essential to diagnosis. Recognition can help provide symptomatic relief and early referral for specialist input and transplantation as needed.
